# Wants

**Published:** 1883-02

**Authors:** 


### Wants.

THE Secretary of the American Dental Association is desirous of completing enough entire series of the Transactions to enable him to place one in each of the principal public libraries of the country, and asks the co-operation of the profession in the work. One dollar each will be paid for one or more copies of the Transactions for the years 1862 and 1863.

GEO. H. CUSHING, Secretary,
34 Monroe Street, Chicago, Ill,

THE fifth annual reunion of the Alumni of the Ohio College of Dental Surgery will take place in the lecture room of the College, Wednesday, March 7th, at 12 o'clock. Banquet at Kepler's same evening at 10 o'clock. E. G. BETTY, Sec'y.

